# Knowledge, Perceptions, and Job Satisfaction of National Emergency Medical Service Employees Regarding Performance Appraisals in Greece: A Comparison of Two Appraisal Systems

**DOI:** 10.7759/cureus.105047

**Published:** 2026-03-11

**Authors:** Panagiota X Ventirozou, Panagiotis Prezerakos, Daphni Kaitelidou, Georgia Kourlaba

**Affiliations:** 1 Department of Nursing, University of the Peloponnese, Tripoli, GRC; 2 Department of Nursing, National and Kapodistrian University of Athens, Athens, GRC

**Keywords:** emergency medical services, employee perceptions, healthcare workforce, health policy, job satisfaction, performance appraisal, public administration, quality improvement

## Abstract

Objective: This study examined the knowledge, perceptions, and job satisfaction of employees of the National Emergency Medical Service (NEMS/EKAB) in Greece regarding two successive performance appraisal systems (Law 4369/2016 and Law 4940/2022).

Methods: A cross-sectional study was conducted across 11 regional branches of the National Emergency Medical Service using stratified proportional sampling. A total of 1,200 employees (paramedics and administrative staff) with at least one year of work experience participated. Data were collected through anonymous, self-administered questionnaires. Knowledge (range: 0-10), perceptions (range: 7-35), and satisfaction (range: 8-40) scores were calculated. Differences between the two appraisal systems were examined using paired statistical tests, while factors associated with knowledge, perceptions, and satisfaction were further explored using multivariable regression models.

Results: The mean knowledge score was significantly higher under the appraisal system of Law 4369/2016 (mean = 7.51, SD = 1.58) compared with Law 4940/2022 (mean = 5.80, SD = 1.90; p < 0.001). In contrast, perceptions were more positive under the newer system (mean = 21.43, SD = 7.38) than under the previous one (mean = 20.62, SD = 7.25; p < 0.001). Similarly, job satisfaction was higher under Law 4940/2022 (mean = 25.05, SD = 8.15) compared with Law 4369/2016 (mean = 23.76, SD = 8.07; p < 0.001). Younger employees and those with fewer years of service reported higher perception and satisfaction scores, whereas greater age and longer work experience were associated with higher knowledge levels but less positive attitudes. Involvement as an evaluator was associated with higher knowledge scores and more positive perceptions, particularly under the Law 4940/2022 system.

Conclusions: Although the earlier appraisal system was better understood by employees, the newer system was perceived as more supportive and was associated with higher job satisfaction. Strengthening training, transparency, and employee participation appears to be critical for improving the acceptance and effectiveness of performance appraisal systems within the National Emergency Medical Service.

## Introduction

Performance appraisal is a fundamental human resource management tool widely used to enhance organizational effectiveness, promote professional development, and ensure the quality of services provided [1]. In the healthcare sector, where staff performance directly affects patient safety, responsiveness, and quality of care, performance appraisal assumes even greater importance [2]. International literature consistently highlights that well-designed appraisal systems contribute to skill development, improved collaboration between employees and management, and increased organizational commitment [3,4].

Performance appraisal systems can also be interpreted through established organizational theories. According to organizational justice theory, employees’ attitudes toward appraisal processes are strongly influenced by perceptions of fairness, transparency, and consistency in evaluation procedures [5,6]. In addition, social exchange theory suggests that fair and supportive appraisal practices enhance trust and reciprocal commitment between employees and the organization [7]. Goal-setting theory further emphasizes the motivational role of clear objectives and constructive feedback, which constitute core components of effective appraisal systems [8,9]. These theoretical perspectives are particularly relevant in high-intensity healthcare environments, where appraisal systems may influence not only administrative outcomes but also professional motivation and service quality [10].

The effectiveness of a performance appraisal system is determined not only by its formal structure but also by employees’ perceptions of fairness, clarity, and transparency throughout the process [5]. When employees perceive appraisal procedures as fair, objective, and meaningful, they tend to exhibit higher levels of job satisfaction, stronger work engagement, and greater motivation to improve performance [6,7,9]. Conversely, perceptions of injustice, lack of transparency, or insufficient feedback may lead to reduced trust, increased occupational stress, and ultimately undermine the intended purpose of the appraisal process [11]. Previous studies have further demonstrated that appraisal satisfaction is closely linked to employee attitudes, motivation, and performance outcomes, particularly within public sector and healthcare organizations [9,12,13].

In the context of emergency prehospital care, such as that provided by the National Emergency Medical Service (NEMS/EKAB) in Greece, performance appraisal assumes particular significance. Employees are required to respond rapidly to emergency situations, collaborate effectively under high-pressure conditions, and operate according to high professional standards. These demands make the systematic assessment, evaluation, and development of staff competencies critically important [9]. In such operational environments, performance appraisal can play a key role in enhancing service quality, operational readiness, and team cohesion, provided that it is implemented consistently and reliably [10].

Despite extensive international research emphasizing the importance of performance appraisal, evidence from the Greek context - particularly within operational healthcare organizations - remains limited and is often non-comparative in nature. Furthermore, recent reforms in public sector performance appraisal systems highlight the need for empirical data regarding employee acceptance, system effectiveness, and potential challenges encountered during implementation [11,12].

Within this framework, the present study aims to systematically investigate the knowledge, perceptions, and job satisfaction of NEMS employees regarding two successive institutional frameworks for public sector performance appraisal. Specifically, the study examines the appraisal system introduced under Law 4369/2016, which was applied until 2022, and the goal-setting and performance appraisal system established by Law 4940/2022 and implemented from 2023 onwards. In addition, demographic and professional factors that may influence employees’ attitudes toward the appraisal process are explored, with the objective of identifying determinants that enhance or hinder the acceptance and effectiveness of performance appraisal systems. By providing updated empirical evidence from a high-intensity operational healthcare setting, this study seeks to contribute to the international discourse on employee appraisal in healthcare organizations and to inform the design of effective performance evaluation systems in demanding operational environments [1,3,10,12].

## Materials and methods

This study was designed as a cross-sectional, quantitative, retrospective comparative study and was conducted across 11 regional branches of the National Emergency Medical Service (NEMS/EKAB) in Greece. Data collection took place between January and April 2025 and aimed to compare employees’ knowledge, perceptions, and job satisfaction regarding two successive performance appraisal systems implemented as part of public administration reforms. The first system was established under Law 4369/2016 and remained in effect until 2022, while the second system was introduced by Law 4940/2022 and has been implemented from 2023 onwards.

Eligible participants were NEMS employees with active employment status (permanent staff or employees with open-ended contracts), belonging to the professional categories of ambulance rescuers/crew members and administrative personnel. Inclusion criteria were: (a) a minimum of one (1) year of service within the organization and (b) participation in the performance appraisal process, either as appraisees or, in a limited number of cases, as appraisers. The role of appraiser was recorded as a distinct variable, irrespective of the participant’s professional category. The requirement of at least one year of service ensured that participants had already undergone the performance appraisal process within the organization. Due to the transitional implementation period between the previous performance appraisal framework (Law 4369/2016) and the newer goal-setting and performance appraisal system (Law 4940/2022), employees included in the study had experience with both appraisal systems. Consequently, participants were able to provide informed responses regarding their knowledge, perceptions, and job satisfaction in relation to each framework.

A stratified proportional sampling method was applied, with stratification based on NEMS regional branches and professional category (ambulance rescuers/crew members and administrative staff). The initial study design aimed to include all 12 NEMS regional branches to ensure maximum geographical representativeness; however, the Thessaloniki branch was excluded due to the absence of administrative approval. Consequently, the study was implemented in the remaining 11 branches.

Following the acquisition of the required institutional approvals from the Research Ethics and Deontology Committee of the University of the Peloponnese and the Scientific Council of the EKAB, the researcher contacted the Directors of the participating branches by telephone to provide detailed information regarding the study’s purpose, procedures, and participation requirements and to coordinate questionnaire distribution. Research materials (printed questionnaires, participant information sheets, and sealed collection envelopes) were subsequently sent to each branch via postal mail.

Within each branch, the Director appointed an authorized staff member as a local contact person responsible for distributing and collecting questionnaires. This individual informed potential participants about the objectives of the study, the voluntary nature of participation, and the assurance of anonymity and confidentiality. The questionnaires were paper-based, self-administered, and distributed in sealed envelopes, which were returned sealed upon completion.

Sample allocation within each branch was proportional to professional category. The required sample size was calculated at 1,249 employees, based on a 95% confidence level and a margin of error of ±2%. Assuming an anticipated response rate of 75%, a total of 1,665 employees were invited to participate. Participation was voluntary, and the final sample included employees who consented and completed the questionnaire.

Completed questionnaires were collected by the designated contact person in each branch and returned to the researcher via postal mail without any identifying information, ensuring full participant anonymity.

Data were collected using printed, self-administered, anonymous questionnaires developed specifically for this study, based on relevant international literature and the legislative framework governing performance appraisal of public sector employees in Greece. The questionnaire consisted of four sections and included a total of 35 items.

The first section included 10 items recording demographic and job-related characteristics, including age, sex, marital status, educational level, professional role, years of service, employment status, grade, supervisory position, and experience in the role of appraiser.

The second section comprised 10 items assessing employees’ knowledge of the performance appraisal processes under Law 4369/2016 and Law 4940/2022. Items addressed appraisal frequency, evaluation criteria, stages of the appraisal process, scoring scales, and appeal procedures. A total knowledge score ranging from 0 to 10 was calculated by assigning one point to each correct answer.

The third section included seven items assessing participants’ perceptions of the performance appraisal process, focusing on perceived usefulness, objectivity, adequacy of information, contribution to professional development, and utilization of appraisal outcomes.

The fourth section consisted of eight items evaluating job satisfaction with the appraisal process, including guidance and support from supervisors, opportunities to express opinions, handling of appeals, and overall satisfaction. Items in sections three and four were rated on five-point Likert scales (1 = strongly disagree to 5 = strongly agree). Perception scores ranged from 7 to 35, and satisfaction scores ranged from 8 to 40.

Content validity was assessed by three independent experts with experience in health services research. Face validity was evaluated with eight NEMS employees. A pilot study involving 41 employees assessed clarity and functionality; pilot responses were excluded from the final analysis. Construct validity was not examined, as the primary objective was comparative evaluation rather than scale development.

Internal consistency reliability was assessed using the Kuder-Richardson Formula 20 (KR-20) for the knowledge section and Cronbach’s alpha for the perceptions and satisfaction sections.

Data were coded and entered into IBM SPSS Statistics version 26 (IBM Corp., Armonk, NY, USA). Statistical analyses were subsequently performed using Stata version 17 (StataCorp LLC, College Station, TX, USA). Descriptive statistics were used to summarize sample characteristics. Associations between categorical variables were examined using Pearson’s chi-square test or Fisher’s exact test, as appropriate. Factors associated with knowledge, perceptions, and satisfaction were examined using multivariable regression models. Statistical significance was set at p < 0.05.

The study adhered to the principles of the Declaration of Helsinki. Ethical approval was obtained from the Research Ethics and Deontology Committee of the University of the Peloponnese (Approval No. 22741/30-10-2024) and from the Scientific Council of the National Emergency Medical Service (EKAB) (Approval No. 50295/29-11-2024). Participation was voluntary, anonymous, and confidential, and informed consent was obtained from all participants.

## Results

Internal consistency reliability indices for the knowledge, perceptions, and satisfaction sections of both questionnaires are presented in Table 1. High internal consistency was observed for the perceptions and satisfaction sections of both appraisal systems, while acceptable reliability levels were identified for the knowledge section.

**Table 1:** Internal consistency reliability indices for the knowledge, perceptions, and satisfaction sections

Section	First questionnaire (Law 4369/2016)	Second questionnaire (Law 4940/2022)
Knowledge	0.53	0.63
Perceptions	0.94	0.95
Satisfaction	0.97	0.98

A total of 1,200 employees of the EKAB participated in the study, corresponding to a response rate of 72.1% (1,200 of 1,665 distributed questionnaires). The distribution of demographic and occupational characteristics is presented in Table 2.

**Table 2:** Demographic and occupational characteristics of the study sample (N = 1,200)

Variable	Category	N	%
Age (years)	≤25	7	0.6
	25–34	29	2.4
	35–44	158	13.2
	45–54	585	48.8
	≥55	421	35.1
Sex	Male	819	68.3
	Female	377	31.4
	Prefer not to answer	4	0.3
Marital status	Married / Civil partnership	901	75.1
	Single	156	13
	Divorced	129	10.8
	Widowed	7	0.6
	Prefer not to answer	7	0.6
Education level	Primary school	5	0.4
	Lower secondary school	8	0.7
	General upper secondary school	660	55
	Vocational upper secondary school	366	30.5
	University / Tertiary education	115	9.6
	Master’s degree	44	3.7
	Doctoral degree	2	0.2
Professional role	Administrative staff	132	11
	Paramedic / Ambulance crew	1065	88.8
	Other	3	0.3
Employment status	Permanent employee	1114	92.8
	Open-ended contract (IDAX)	86	7.2
Grade	Grade A	864	72
	Grade B	133	11.1
	Grade C	107	8.9
	Grade D	62	5.2
	Grade E	34	2.8
Position	Director	3	0.3
	Head of Department	5	0.4
	Acting Head of Department	7	0.6
	Sector Supervisor	25	2.1
	Employee	1160	96.7
Appraiser role	No	1187	98.9
	Yes	13	1.1

Nearly half of the participants were aged 45-54 years (48.8%), while 35.1% were aged 55 years or older. The majority of respondents were male (68.3%) and married or in a civil partnership (75.1%). Regarding educational attainment, 55% were graduates of General Upper Secondary School and 30.5% of Technical and Vocational Upper Secondary School.

Most participants were employed as ambulance rescuers or crew members (88.8%), while 11% were administrative staff. The vast majority were permanent employees (92.8%). With respect to employment grade, 72% held Grade A positions, and 96.7% occupied staff-level positions rather than supervisory roles.

The overall knowledge score was significantly higher for the appraisal system established under Law 4369/2016 (mean = 7.51, SD = 1.58) compared with the system introduced by Law 4940/2022 (mean = 5.80, SD = 1.90), with the difference reaching statistical significance (p < 0.001). This finding indicates greater employee familiarity with the earlier performance appraisal framework.

At the level of individual knowledge items, the appraisal system under Law 4369/2016 demonstrated particularly high rates of correct responses for core and frequently applied components of the appraisal process. These included appraisal frequency (96.6%), identification of the responsible evaluator (96.3%), the scoring scale (87.3%), and the consequences of a negative appraisal (92.2%). These findings suggest adequate employee knowledge of the central and operational aspects of the earlier system.

However, notable knowledge gaps were identified for more complex or less prominently communicated procedural elements. Only 33.1% of participants correctly identified all formal stages of the appraisal process, while 40.8% correctly identified the conditions under which an appeal could be submitted.

In contrast, the appraisal system introduced by Law 4940/2022 exhibited more extensive and pronounced knowledge gaps. Although high proportions of correct responses were observed for identifying the responsible evaluator (95.4%) and the consequences of a negative appraisal (92.9%), knowledge of key functional characteristics was limited. Only 12.9% of participants correctly identified that performance appraisal is conducted twice annually, and just 46.1% were aware of the correct scoring scale (1-5).

Furthermore, low rates of correct responses were recorded regarding procedural aspects of the new system, including the conditions for submitting an appeal (38.3%) and the content of the appraisal report (54.5%). These findings suggest substantial confusion surrounding critical components of the newer institutional framework.

Overall, employees demonstrated satisfactory knowledge of the general principles and core structures of performance appraisal systems; however, significant knowledge gaps persisted regarding implementation details, particularly under the appraisal system introduced by Law 4940/2022. These gaps may negatively affect employees’ understanding, acceptance, and effective engagement with the appraisal process (Table 3).

**Table 3:** Detailed responses on knowledge of the performance appraisal process NEMS: National Emergency Medical Service

Question	Response option	Law 4369/2016N (%)	Law 4940/2022N (%)
How often is the appraisal conducted?	Once per year	1159 (96.6)	1005 (83.8)
	Once per month	9 (0.8)	40 (3.3)
	Twice per year	32 (2.7)	155 (12.9)
What are the main objectives of the appraisal system as defined by law?	Improving the functioning of public sector services and organizations	498 (41.5)	411 (34.3)
	Improving individual employee performance and overall public service performance	681 (56.8)	761 (63.4)
	Increasing public employees’ salaries	21 (1.8)	28 (2.3)
What are the main appraisal criteria for public sector employees according to the law?	Job knowledge, initiative and creativity, professional relationships and behavior, effectiveness	794 (66.2)	535 (44.6)
	Citizen orientation, teamwork, adaptability, results orientation, organization and planning, problem solving	313 (26.1)	600 (50)
	Effectiveness	93 (7.8)	65 (5.4)
Who is responsible for conducting the appraisal according to the law?	Department Head and Directorate Head	1156 (96.3)	1145 (95.4)
	Independent appraisal committee	35 (2.9)	47 (3.9)
	External consultant	9 (0.8)	8 (0.7)
What are the stages of the appraisal process as defined by law?	Development plan, progress review, team pulse survey, and overall appraisal	397 (33.1)	749 (62.4)
	Appraisal by supervisors	749 (62.4)	404 (33.7)
	Self-appraisal	54 (4.5)	47 (3.9)
What is the scoring scale according to the law?	1–100	1048 (87.3)	604 (50.3)
	1–5	114 (9.5)	553 (46.1)
	1–1,000	38 (3.2)	43 (3.6)
When is an employee entitled to file an appeal according to the law?	If they disagree with their score	632 (52.7)	548 (45.7)
	If their total score does not exceed 2	79 (6.6)	460 (38.3)
	If the average score is below 75	489 (40.8)	192 (16)
What does the appraisal report include according to the law?	Appraisee’s score, mandatory selection of three skills for development and one to three developed skills, and a special section	273 (22.8)	654 (54.5)
	Educational qualifications, training activities, and summary description of work performed by the organizational unit	775 (64.6)	478 (39.8)
	Score and comments	152 (12.7)	68 (5.7)
What are the consequences of a negative appraisal according to the law?	Provision of additional training and support	1106 (92.2)	1115 (92.9)
	Automatic dismissal	36 (3)	49 (4.1)
	Salary reduction	58 (4.8)	36 (3)
Are there special provisions for NEMS staff according to the law?	The law applies generally, with no special provisions for NEMS	1057 (88.1)	1075 (89.6)
	There are special provisions only for NEMS employees	108 (9)	84 (7)
	The law does not apply to NEMS	35 (2.9)	41 (3.4)

The overall perceptions score was significantly higher under the appraisal system of Law 4940/2022 (mean = 21.43, SD = 7.38) compared with Law 4369/2016 (mean = 20.62, SD = 7.25), with the difference reaching statistical significance (p < 0.001). This finding suggests a modest enhancement in employees’ positive attitudes toward the newer appraisal system.

Regarding the overall perceived usefulness of the appraisal process, the majority of participants in both periods characterized the process as useful or very useful (50.4% for Law 4369/2016 and 47.5% for Law 4940/2022). However, a higher proportion of neutral responses was observed under the newer system (26% vs. 20.9%), indicating uncertainty or lack of a clearly formed stance toward the appraisal process.

With respect to perceptions of fairness and objectivity, a moderate improvement was observed under Law 4940/2022, as the proportion of respondents reporting agreement or strong agreement increased from 29.8% under the earlier system to 33.7% under the newer one. At the same time, rates of strong disagreement declined, although the proportion of neutral responses remained high (39.5%), reflecting ongoing reservations regarding the reliability and transparency of the process.

Concerning the level of information provided about appraisal criteria, a slight improvement was recorded under the newer system, with the proportion of employees reporting that they felt informed (agree or strongly agree) increasing from 31.8% to 34.8%. Nevertheless, more than one-third of participants in both periods maintained a neutral position, suggesting that the information provided may not be sufficiently clear or comprehensible to all employees.

With regard to the contribution of appraisal to professional development, a small but clear improvement was observed under Law 4940/2022, as the proportion of strong agreement increased from 9% to 11.2%. Despite this improvement, a substantial proportion of participants (34.8%) expressed a neutral stance, indicating that the link between appraisal and professional development is not clearly perceived by all employees.

In terms of the use of appraisal results to improve performance, approximately one-third of employees in both periods reported that they frequently or always use appraisal outcomes. However, a considerable proportion indicated that they rarely or never use appraisal results, suggesting limited functional integration of the appraisal process into everyday professional practice.

Finally, regarding perceptions that appraisal results are used for recognition and reward, and that appraisals accurately reflect actual performance, a clear improvement was observed under the newer system, with the proportion of strong agreement nearly doubling in both cases. Despite this improvement, the predominant response remained neutral, indicating limited trust in the linkage between appraisal, recognition, reward, and objective performance assessment (Table 4).

**Table 4:** Personal perceptions regarding the performance appraisal process at National Emergency Medical Service (NEMS/EKAB)

Statement	Response option	Law 4369/2016N (%)	Law 4940/2022N (%)
Do you believe that the appraisal process is:	Not useful at all	228 (19)	190 (15.8)
	Slightly useful	116 (9.7)	128 (10.7)
	Neutral	251 (20.9)	312 (26)
	Useful	378 (31.5)	358 (29.8)
	Very useful	227 (18.9)	212 (17.7)
The appraisal process is fair and objective	Strongly disagree	180 (15)	142 (11.8)
	Disagree	240 (20)	179 (14.9)
	Neither agree nor disagree	422 (35.2)	474 (39.5)
	Agree	282 (23.5)	293 (24.4)
	Strongly agree	76 (6.3)	112 (9.3)
I feel well-informed about the appraisal criteria used	Strongly disagree	127 (10.6)	141 (11.8)
	Disagree	256 (21.3)	199 (16.6)
	Neither agree nor disagree	436 (36.3)	443 (36.9)
	Agree	303 (25.3)	306 (25.5)
	Strongly agree	78 (6.5)	111 (9.3)
The appraisal contributes to my professional development	Strongly disagree	151 (12.6)	163 (13.6)
	Disagree	265 (22.1)	186 (15.5)
	Neither agree nor disagree	360 (30)	418 (34.8)
	Agree	316 (26.3)	299 (24.9)
	Strongly agree	108 (9)	134 (11.2)
I use my appraisal results to improve my performance	Never	270 (22.5)	248 (20.7)
	Rarely	189 (15.8)	155 (12.9)
	Sometimes	299 (24.9)	341 (28.4)
	Often	237 (19.8)	250 (20.8)
	Always	205 (17.1)	206 (17.2)
Appraisal results are used to recognize and reward my efforts	Strongly disagree	210 (17.5)	135 (11.3)
	Disagree	245 (20.4)	219 (18.3)
	Neither agree nor disagree	361 (30.1)	439 (36.6)
	Agree	303 (25.3)	272 (22.7)
	Strongly agree	81 (6.8)	135 (11.3)
The appraisals I receive are accurate and reflect my actual performance	Strongly disagree	201 (16.8)	153 (12.8)
	Disagree	225 (18.8)	198 (16.5)
	Neither agree nor disagree	411 (34.3)	452 (37.7)
	Agree	277 (23.1)	270 (22.5)
	Strongly agree	86 (7.2)	127 (10.6)

The overall satisfaction score was significantly higher under the appraisal system of Law 4940/2022 (mean = 25.05, SD = 8.15) compared with Law 4369/2016 (mean = 23.76, SD = 8.07; p < 0.001), indicating an overall improvement in employees’ appraisal-related experience under the newer institutional framework.

Across specific dimensions of satisfaction, the system of Law 4940/2022 showed increased rates of strong agreement regarding guidance provided by the service (10% vs. 7.8%) and support from supervisors during the appraisal process (10.9% vs. 8.4%). In parallel, a clear improvement was observed in employees’ perceived opportunity to express their views and highlight their competencies, with the proportion of strong agreement increasing from 7.4% under the earlier system to 11.3% under the newer one.

A similar positive shift was noted with respect to the time allocated to the appraisal interview, where strong agreement increased from 8.2% to 11.3%, indicating improvement in the procedural dimension of the appraisal process. Additionally, a modest improvement was observed in satisfaction with the appraisal received, with strong agreement increasing from 9.3% under Law 4369/2016 to 11.1% under Law 4940/2022.

Regarding satisfaction with how supervisors take employees’ feedback into account, as well as with the management of appeals and disagreements, improvements were also evident under the newer system. Specifically, strong agreement increased from 8.7% to 10.3% and from 5.7% to 9.3%, respectively. Despite these improvements, high proportions of neutral responses persisted, indicating that feedback mechanisms and dispute resolution processes are not yet fully established.

Finally, with respect to overall satisfaction with the appraisal process currently implemented at EKAB, a clear increase in strong agreement was observed under the newer system (10% vs. 6.4%). Nevertheless, the overall picture remains mixed, as a substantial proportion of employees maintained a neutral stance (40.8%), suggesting that despite incremental improvements, the appraisal process is not perceived as uniformly positive across the workforce (Table 5).

**Table 5:** Job satisfaction with the performance appraisal process at National Emergency Medical Service (NEMS/EKAB)

Statement	Response option	Law 4369/2016N (%)	Law 4940/2022N (%)
I am satisfied with the guidance I receive from my service regarding the appraisal process	Strongly disagree	161 (13.4)	124 (10.3)
	Disagree	252 (21)	187 (15.6)
	Neither agree nor disagree	392 (32.7)	455 (37.9)
	Agree	301 (25.1)	314 (26.2)
	Strongly agree	94 (7.8)	120 (10)
I am satisfied with the support I receive from my supervisor during the appraisal process	Strongly disagree	149 (12.4)	133 (11.1)
	Disagree	212 (17.7)	150 (12.5)
	Neither agree nor disagree	402 (33.5)	452 (37.7)
	Agree	336 (28)	334 (27.8)
	Strongly agree	101 (8.4)	131 (10.9)
I am satisfied with the opportunity provided during the appraisal process to express my views and demonstrate my abilities	Strongly disagree	152 (12.7)	149 (12.4)
	Disagree	252 (21)	158 (13.2)
	Neither agree nor disagree	399 (33.3)	453 (37.8)
	Agree	308 (25.7)	304 (25.3)
	Strongly agree	89 (7.4)	136 (11.3)
I am satisfied with the time allocated to the appraisal interview	Strongly disagree	151 (12.6)	136 (11.3)
	Disagree	216 (18)	166 (13.8)
	Neither agree nor disagree	434 (36.2)	451 (37.6)
	Agree	301 (25.1)	311 (25.9)
	Strongly agree	98 (8.2)	136 (11.3)
I am satisfied with the appraisal I received in 2022 or 2023, respectively	Strongly disagree	128 (10.7)	111 (9.3)
	Disagree	183 (15.3)	168 (14)
	Neither agree nor disagree	462 (38.5)	476 (39.7)
	Agree	315 (26.3)	312 (26)
	Strongly agree	112 (9.3)	133 (11.1)
I am satisfied that my supervisors take my feedback into account during the appraisal process	Strongly disagree	140 (11.7)	117 (9.8)
	Disagree	179 (14.9)	165 (13.8)
	Neither agree nor disagree	431 (35.9)	452 (37.7)
	Agree	346 (28.8)	342 (28.5)
	Strongly agree	104 (8.7)	124 (10.3)
I am satisfied with the way appeals and disagreements are handled during the appraisal process	Strongly disagree	145 (12.1)	116 (9.7)
	Disagree	200 (16.7)	173 (14.4)
	Neither agree nor disagree	496 (41.3)	497 (41.4)
	Agree	291 (24.3)	302 (25.2)
	Strongly agree	68 (5.7)	112 (9.3)
Overall, I am satisfied with the performance appraisal process currently implemented at NEMS	Strongly disagree	206 (17.2)	96 (8)
	Disagree	253 (21.1)	174 (14.5)
	Neither agree nor disagree	421 (35.1)	490 (40.8)
	Agree	243 (20.3)	320 (26.7)
	Strongly agree	77 (6.4)	120 (10)

Under the performance appraisal system of Law 4369/2016, univariate analyses showed that higher knowledge levels were statistically significantly associated with older age, longer years of service, and permanent employment status, whereas lower knowledge scores were observed among unmarried employees, those holding lower hierarchical grades, and those without managerial positions. In the multivariable analysis, age, marital status, length of service, employment status, and grade remained independently and statistically significantly associated with knowledge scores (Table 6).

**Table 6:** Univariate and multivariate linear regression analysis for the knowledge score to the first questionnaire (Law 4369/2016)

Independent variables	Univariate coefficient (95% CI)	p-value	Multivariable coefficient (95% CI)	p-value
Age, years				
35–44 vs. <35	0.83 (0.26 to 1.40)	0.004	0.33 (-0.29 to 0.95)	0.3
45–54 vs. <35	1.21 (0.68 to 1.73)	<0.001	0.54 (-0.09 to 1.16)	0.091
≥55 vs. <35	1.45 (0.92 to 1.98)	<0.001	0.91 (0.25 to 1.56)	0.007
Sex				
Female vs. Male	0.14 (-0.06 to 0.33)	0.159	0.19 (-0.01 to 0.38)	0.062
Marital status				
Single vs. Married/Civil partnership	-0.94 (-1.12 to -0.67)	<0.001	-0.71 (-1.00 to -0.43)	<0.001
Divorced/Widowed vs. Married/Civil partnership	-0.15 (-0.43 to 0.13)	0.306	-0.19 (-0.47 to 0.09)	0.182
Education				
General high school vs. Primary/Lower secondary	-0.52 (-1.39 to 0.35)	0.240	0.70 (-0.15 to 1.56)	0.105
Vocational high school vs. Primary/Lower secondary	-0.51 (-1.39 to 0.36)	0.251	0.73 (-0.13 to 1.58)	0.098
University/Postgraduate/PhD vs. Primary/Lower secondary	-0.51 (-1.41 to 0.39)	0.265	0.39 (-0.50 to 1.28)	0.387
Professional role				
Paramedics/Ambulance crew vs. Supervisor/Unit head	-0.21 (-0.50 to 0.07)	0.146	-0.41 (-0.76 to -0.05)	0.024
Other vs. Supervisor/Unit head	1.30 (-0.51 to 3.12)	0.158	0.41 (-1.59 to 2.41)	0.685
Years of service (per year) (per year)	0.02 (0.01 to 0.03)	<0.001	-0.03 (-0.05 to 0.00)	0.022
Employment status				
Open-ended contract vs. Permanent employee	1.64 (0.03 to 3.26)	0.047	-0.47 (-0.83 to -0.10)	0.013
Grade				
B vs. A	-0.24 (-0.53 to 0.05)	0.103	-0.23 (-0.56 to 0.10)	0.174
C vs. A	-0.35 (-0.66 to -0.03)	0.030	-0.33 (-0.74 to 0.07)	0.107
D vs. A	-0.60 (-1.00 to -0.19)	0.004	-0.46 (-1.01 to 0.08)	0.093
E vs. A	-1.04 (-1.58 to -0.50)	<0.001	-0.83 (-1.53 to -0.12)	0.021
Position				
Employee vs. Supervisor/Unit head	-0.51 (-1.00 to -0.01)	0.047	-0.29 (-0.83 to 0.25)	0.294
Evaluator role				
Yes vs. No	0.81 (-0.06 to 1.67)	0.068	0.29 (-0.75 to 1.32)	0.589

For the appraisal system introduced by Law 4940/2022, univariate analyses indicated lower knowledge levels among ambulance rescuers/crew members, while higher knowledge scores were observed among employees with experience in the role of evaluator. In the multivariable analysis, professional role remained the sole factor statistically significantly associated with knowledge scores (Table 7).

**Table 7:** Univariate and multivariable linear regression analysis for the knowledge score to the second questionnaire (Law 4940/2022).

Independent variables	Univariate models		Multivariable model	
	N=1,200		N=1,193	
	Coefficient (95% CI)	p-value	Coefficient (95% CI)	p-value
Age (years)				
35–44 vs. <35	0.12 (-0.61 to 0.84)	0.755	0.12 (-0.61 to 0.84)	0.755
45–54 vs. <35	0.35 (-0.33 to 1.03)	0.309	0.35 (-0.33 to 1.03)	0.309
≥55 vs. <35	0.10 (-0.58 to 0.79)	0.769	0.10 (-0.58 to 0.79)	0.769
Sex	N=1,196		N=1,196	
Female vs. Male	-0.44 (-0.36 to 0.13)	0.364	-0.44 (-0.36 to 0.13)	0.364
Marital status	N=1,193		N=1,193	
Single vs. Married/Civil partnership	-0.34 (-0.68 to 0.01)	0.050	-0.34 (-0.68 to 0.01)	0.050
Divorced/Widowed vs. Married/Civil partnership	-0.10 (-0.47 to 0.26)	0.578	-0.10 (-0.47 to 0.26)	0.578
Education level	N=1,200		N=1,200	
General high school vs. Primary/Lower secondary education	-0.39 (-1.49 to 0.71)	0.485	-0.39 (-1.49 to 0.71)	0.485
Vocational high school vs. Primary/Lower secondary education	-0.25 (-1.36 to 0.86)	0.667	-0.25 (-1.36 to 0.86)	0.667
University/Tertiary, Postgraduate, or Doctoral degree vs. Primary/Lower secondary education	0.29 (-0.84 to 1.42)	0.617	0.29 (-0.84 to 1.42)	0.617
Professional role	N=1,200		N=1,200	
Paramedics/Ambulance crew vs. Supervisor/Unit head	-0.86 (-1.22 to -0.49)	<0.001	-0.86 (-1.22 to -0.49)	<0.001
Other vs. Supervisor/Unit head	1.52 (-0.76 to 3.80)	0.191	1.52 (-0.76 to 3.80)	0.191
Years of service (per year)	N=1,200		N=1,200	
	-0.01 (-0.03 to 0.01)	0.198	-0.01 (-0.03 to 0.01)	0.198
Employment status	N=1,200		N=1,200	
Open-ended contract vs. Permanent employee	0.44 (0.02 to 0.86)	0.04	0.44 (0.02 to 0.86)	0.04
Rank	N=1,200		N=1,200	
Β vs. Α	0.38 (0.01 to 0.75)	0.042	0.38 (0.01 to 0.75)	0.042
C vs. Α	0.18 (-0.22 to 0.59)	0.376	0.18 (-0.22 to 0.59)	0.376
D vs. Α	-0.24 (-0.76 to 0.28)	0.369	-0.24 (-0.76 to 0.28)	0.369
Ε vs. Α	0.50 (-0.18 to 1.19)	0.151	0.50 (-0.18 to 1.19)	0.151
Position	N=1,200		N=1,200	
Employee vs. Supervisor/Unit head	-0.23 (-0.86 to 0.41)	0.483	-0.23 (-0.86 to 0.41)	0.483
Evaluator role	N=1,200		N=1,200	
Yes vs. No	1.11 (0.01 to 2.21)	0.048	1.11 (0.01 to 2.21)	0.048

Analysis of changes in knowledge scores between the two appraisal systems demonstrated a greater decline in knowledge among older employees, those with longer years of service, and ambulance rescuers, whereas a smaller decline was observed among employees with permanent employment contracts and those holding lower hierarchical grades. In the multivariable analysis, employment status and grade emerged as independent factors statistically significantly associated with changes in knowledge scores (Table 8).

**Table 8:** Regression analysis for the change in knowledge score between the two appraisal systems

Independent variables	Univariate coefficient (95% CI)	p-value	Multivariable coefficient (95% CI)	p-value
Age (years)				
35–44 vs. <35	-0.72 (-1.58 to 0.14)	0.101	-0.37 (-1.32 to 0.58)	0.444
45–54 vs. <35	-0.85 (-1.65 to -0.06)	0.036	-0.34 (-1.29 to 0.61)	0.481
≥55 vs. <35	-1.35 (-2.15 to -0.54)	0.001	-0.93 (-1.92 to 0.07)	0.069
Sex				
Female vs. Male	-0.25 (-0.54 to 0.04)	0.089	-0.47 (-0.77 to 0.17)	0.092
Marital status				
Single vs. Married/Civil partnership	0.59 (0.19 to 1.00)	0.004	0.38 (-0.06 to 0.81)	0.088
Divorced/Widowed vs. Married/Civil partnership	0.04 (-0.39 to 0.47)	0.843	0.07 (-0.36 to 0.50)	0.755
Education level				
General high school vs. Primary/Lower secondary	-0.91 (-2.22 to 0.39)	0.170	-0.78 (-2.08 to 0.52)	0.237
Technical vocational high school vs. Primary/Lower secondary	-0.76 (-2.08 to 0.55)	0.256	-0.65 (-1.96 to 0.66)	0.331
University/Postgraduate/PhD vs. Primary/Lower secondary	-0.22 (-1.56 to 1.12)	0.747	-0.17 (-1.52 to 1.18)	0.808
Professional role				
Paramedics/Ambulance crew vs. Supervisor/Unit head	-0.64 (-1.07 to -0.21)	0.003	-0.35 (-0.89 to 0.19)	0.200
Other vs. Supervisor/Unit head	0.22 (-2.50 to 2.94)	0.874	0.76 (-2.29 to 3.81)	0.625
Years of service (per year)	-0.03 (-0.05 to -0.01)	<0.001	0.03 (-0.01 to 0.06)	0.147
Employment status				
Open-ended contract vs. Permanent employee	1.11 (0.59 to 1.63)	<0.001	0.78 (0.22 to 1.34)	0.006
Grade				
B vs. A	0.62 (0.19 to 1.05)	0.005	0.61 (0.11 to 1.12)	0.017
C vs. A	0.53 (0.06 to 1.01)	0.028	0.57 (-0.05 to 1.19)	0.071
D vs. A	0.36 (-0.25 to 0.97)	0.247	0.20 (-0.63 to 1.02)	0.642
E vs. A	1.55 (0.74 to 2.36)	<0.001	1.35 (0.28 to 2.43)	0.013
Position				
Employee vs. Supervisor/Unit head	0.28 (-0.47 to 1.03)	0.468	0.50 (-0.33 to 1.32)	0.238
Evaluator role				
Yes vs. No	0.31 (-0.99 to 1.61)	0.646	0.06 (-1.52 to 1.64)	0.937

For the appraisal system of Law 4369/2016, less positive perceptions of the appraisal process were associated with older age and longer years of service, whereas more positive perceptions were observed among unmarried employees, those holding higher hierarchical grades, and those who had previously served as evaluators. Additionally, a negative association was identified between knowledge scores and positive perceptions of the appraisal process. In the multivariable analysis, marital status, grade, and knowledge score remained statistically significant factors (Table 9).

**Table 9:** Univariate and multivariable linear regression analysis for perception score – first questionnaire (Law 4369/2016)

Independent variables	Univariate models coefficient (95% CI)	p-value	Multivariable model coefficient (95% CI)	p-value
Age (years)				
35–44 vs. <35	-3.27 (-5.92 to -0.61)	0.014	-1.10 (-3.98 to 1.77)	0.452
45–54 vs. <35	-4.34 (-6.81 to -1.88)	<0.001	-2.21 (-5.11 to 0.69)	0.135
≥55 vs. <35	-4.58 (-7.07 to -2.08)	<0.001	-2.31 (-5.36 to 0.73)	0.137
Sex				
Female vs. Male	0.02 (-0.87 to 0.92)	0.957	-0.05 (-0.97 to 0.86)	0.909
Marital status				
Single vs. Married/Civil partnership	3.03 (1.79 to 4.28)	<0.001	2.48 (1.15 to 3.81)	<0.001
Divorced/Widowed vs. Married/Civil partnership	1.05 (-0.27 to 2.37)	0.08	1.04 (-0.26 to 2.35)	0.117
Education level				
General High School vs. Primary/Lower secondary	-2.84 (-6.89 to 1.20)	0.148	-3.37 (-7.33 to 0.58)	0.095
Vocational High School vs. Primary/Lower secondary	-3.16 (-7.24 to 0.91)	0.110	-3.76 (-7.75 to 0.22)	0.064
University/Postgraduate/PhD vs. Primary/Lower secondary	-2.27 (-6.43 to 1.90)	0.278	-3.39 (-7.50 to 0.72)	0.106
Professional role				
Paramedics/Crew vs. Supervisor/Unit head	-0.52 (-1.85 to 0.81)	0.360	1.41 (-7.86 to 10.69)	0.765
Other vs. Supervisor/Unit head	6.18 (-2.25 to 14.61)	0.144	—	—
Years of service (per year)	-0.06 (-0.11 to -0.01)	0.047	-0.07 (-0.17 to 0.04)	0.204
Employment status				
Open-ended contract vs. Permanent	0.95 (-0.66 to 2.57)	0.247	0.16 (-1.54 to 1.87)	0.85
Grade				
B vs. A	-0.49 (-1.83 to 0.84)	0.573	-1.34 (-2.87 to 0.19)	0.086
C vs. A	-0.27 (-1.74 to 1.21)	0.837	-2.07 (-3.96 to -0.18)	0.032
D vs. A	-0.79 (-2.68 to 1.10)	0.474	-3.44 (-5.95 to -0.93)	0.007
E vs. A	4.54 (2.02 to 7.05)	<0.001	1.48 (-1.79 to 4.74)	0.376
Position				
Employee vs. Supervisor/Unit head	-4.08 (-6.39 to -1.77)	0.001	-1.28 (-3.78 to 1.23)	0.318
Evaluator role				
Yes vs. No	5.51 (1.49 to 9.52)	0.007	4.76 (-0.05 to 9.57)	0.052
Knowledge score	-0.47 (-0.73 to -0.22)	<0.001	-0.33 (-0.60 to -0.07)	0.014

Similarly, for the appraisal system of Law 4940/2022, lower levels of positive perceptions were associated with older age and lower hierarchical position, whereas higher knowledge levels and experience in the role of evaluator were associated with more positive attitudes toward the new appraisal system (Table 10).

**Table 10:** Univariate and multivariable linear regression analysis for perception score – second questionnaire (Law 4940/2022)

Independent variables	Univariate models coefficient (95% CI)	p-value	Multivariable models coefficient (95% CI)	p-value
Age (years)				
35–44 vs. <35	-2.59 (-5.26 to 0.08)	0.057	-0.82 (-3.77 to 2.13)	0.587
45–54 vs. <35	-3.19 (-5.67 to -0.71)	0.012	-1.19 (-4.16 to 1.78)	0.431
≥55 vs. <35	-3.35 (-5.86 to -0.84)	0.009	-1.12 (-4.24 to 1.99)	0.479
Sex				
Female vs. Male	0.34 (-0.56 to 1.24)	0.455	0.30 (-0.63 to 1.24)	0.524
Marital status				
Single vs. Married/Civil partnership	2.27 (1.02 to 3.52)	<0.001	2.17 (0.81 to 3.52)	0.002
Divorced/Widowed vs. Married/Civil partnership	0.46 (-0.87 to 1.79)	0.496	0.43 (-0.91 to 1.76)	0.532
Education				
General High School vs. Primary/Lower Secondary	-2.88 (-6.93 to 1.17)	0.163	-3.14 (-7.19 to 0.91)	0.129
Vocational High School vs. Primary/Lower Secondary	-3.68 (-7.76 to 0.40)	0.077	-4.11 (-8.19 to 0.03)	0.058
University/Postgraduate/PhD vs. Primary/Lower Secondary	-2.42 (-6.59 to 1.74)	0.254	-3.02 (-7.23 to 1.20)	0.160
Professional role				
Paramedics/Ambulance crew vs. Supervisor/Unit head	-0.36 (-1.69 to 0.98)	0.599	0.73 (-0.96 to 2.42)	0.396
Other vs. Supervisor/Unit head	10.28 (1.85 to 18.71)	0.017	5.26 (-4.26 to 14.77)	0.278
Years of service (per year)	-0.06 (-0.11 to 0.001)	0.055	-0.01 (-0.12 to 0.09)	0.783
Employment status				
Open-ended contract vs. Permanent employee	1.64 (0.03 to 3.26)	0.047	1.11 (-0.64 to 2.86)	0.213
Grade				
B vs. A	-0.07 (-1.42 to 1.27)	0.917	-0.63 (-2.20 to 0.94)	0.428
C vs. A	0.73 (-0.76 to 2.21)	0.337	-0.21 (-2.14 to 1.72)	0.831
D vs. A	0.00 (-1.90 to 1.90)	1	-1.30 (-3.87 to 1.27)	0.322
E vs. A	3.43 (0.91 to 5.96)	0.008	1.55 (-1.79 to 4.90)	0.362
Position				
Employee vs. Supervisor/Unit head	-1.91 (-4.24 to 0.42)	0.107	-0.53 (-3.10 to 2.03)	0.683
Evaluator role				
Yes vs. No	6.10 (2.08 to 10.12)	0.003	4.69 (-0.23 to 9.62)	0.062
Knowledge score	0.42 (0.21 to 0.63)	<0.001	0.42 (0.21 to 0.63)	<0.001

Satisfaction with the performance appraisal process was statistically significantly associated with the change in employees’ knowledge scores, as well as with certain occupational characteristics. In the multivariable regression model, higher satisfaction levels were observed among employees with open-ended contracts compared with permanent employees and among those in Grade C compared with Grade A. In addition, a greater increase in knowledge scores was significantly associated with higher satisfaction with the appraisal process (Table 11).

**Table 11:** Regression analysis for satisfaction score with the performance appraisal process

Independent variables	Univariate models coefficient (95% CI)	p-value	Multivariable model coefficient (95% CI)	p-value
Age (years)	N=1.200			
35–44 vs. <35	-0.50 (-2.68 to 1.69)	0.657	0.23 (-2.19 to 2.64)	0.852
45-54 vs. < 35	-0.28 (-2.31 to 1.75)	0.787	1.2 (-1.23 to 3.62)	0.335
≥55 vs. <35	0.14 (-1.91 to 2.20)	0.908	2.13 (-0.42 to 4.68)	0.101
Sex	N=1.196			
Female vs. Male	-0.12 (-0.85 to 0.62)	0.76	0.12 (-0.64 to 0.89)	0.753
Marital status	N=1.193			
Single vs. Married/Civil partnership	0.21 (-0.81 to 1.24)	0.67	0.05 (-1.06 to 1.15)	0.935
Divorced/Widowed vs. Married/Civil partnership	-0.23 (-1.32 to 0.85)	0.689	-0,29 (-1,39 to 0,8)	0.598
Education	N=1.200			
General high school vs. Primary/Lower secondary education	-0.70 (-4.01 to 2.61)	0.674	0.39 (-2.93 to 3.71)	0.818
Vocational high school vs. Primary/Lower secondary education	-0.89 (-4.22 to 2.45)	0.03	0.07 (-3.27 to 3.41)	0.968
University/Tertiary education – Postgraduate – Doctoral degree vs. Primary/Lower secondary education	-0.97 (-4.38 to 2.44)	0.577	0.23 (-3.22 to 3.68)	0.895
Professional role	N=1.200			
Paramedics/Ambulance crew vs. Supervisor/Unit head	0.12 (-0.97 to 1.21)	0.842	0.52 (-0.85 to 1.9)	0.456
Other vs. Supervisor/Unit head	4.83 (-2.07 to 11.73)	0.171	4.22 (-3.56 to 12.01)	0.287
Years of service	N=1.200			
	-0.04 (-0.09 to 0.01)	0.077	-0.01 (-0.1 to 0.07)	0.78
Employment status	N=1.200			
Open-ended contract vs. Permanent employee	2.06 (0.75 to 3.38)	0.002	1.7 (0.27 to 3.14)	0.02
Grade	N=1.200			
Β vs. Α	0.81 (-0.29 to 1.90)	0.149	0.86 (-0.43 to 2.14)	0.192
C vs. Α	1.60 (0.39 to 2.81)	0.012	1.8 (0.22 to 3.38)	0.026
D vs. Α	1.75 (0.20 to 3.30)	0.027	1.79 (-0.31 to 3.9)	0.095
Ε vs. Α	0.30 (-1.76 to 2.36)	0.771	-0.25 (-2.99 to 2.49)	0.859
Position	N=1.200			
Employee vs. Supervisor/Unit head	-0.03 (-1.93 to 1.87)	0.972	0.16 (-1.94 to 2.27)	0.878
Evaluator role	N=1.200			
Yes vs. No	1.18 (-2.11 to 4.48)	0.482	0.53 (-3.5 to 4.56)	0.797
Change in knowledge score (Δ knowledge score)	N=1.200			
	0.34 (0.19 to 0.48)	<0.001	0.34 (0.19 to 0.48)	<0.001

## Discussion

The present study examined employees’ knowledge, perceptions, and levels of satisfaction regarding two consecutive performance appraisal systems implemented at the EKAB. The findings indicate that, although the earlier appraisal system was more familiar to employees, the newer system was associated with more positive perceptions and higher levels of satisfaction. This result suggests that the quality, functionality, and perceived fairness of the appraisal process outweigh mere familiarity with the system. Although increased familiarity might be expected to enhance acceptance, the findings indicate that employees place greater value on appraisal systems that are perceived as fair, transparent, and meaningful, regardless of how long they have been in place. This may reflect a critical stance among employees in high-demand healthcare settings, where appraisal processes are evaluated primarily on their practical relevance, credibility, and contribution to professional development rather than on habituation or routine exposure.

This finding is consistent with the international literature, which emphasizes that acceptance of performance appraisal systems largely depends on their perceived reliability, transparency, and fairness rather than on their long-term implementation [1,8]. In this context, the higher satisfaction observed with the newer appraisal system, despite lower levels of knowledge, suggests that employees evaluate more positively appraisal processes that are perceived as more supportive and meaningful for their professional development.

Of particular interest is the finding that younger employees and those with fewer years of service exhibited more positive perceptions and higher levels of satisfaction with the appraisal process. This pattern may be attributed to greater adaptability to organizational change, as well as to differing expectations from the organization, since younger generations of employees tend to place greater value on feedback, participation, and continuous skill development. In contrast, employees with longer tenure appeared to approach changes in appraisal systems with greater reservation, possibly due to previous experiences or limited access to adequate information.

The study findings also highlight the importance of information provision and active employee involvement in the appraisal process. Limited knowledge of the new appraisal system was associated with less positive attitudes, underscoring the need for systematic communication, training, and the provision of clear and accessible information. International evidence supports that employee participation and high-quality feedback enhance trust in appraisal systems and contribute to the development of positive perceptions [9-11].

In healthcare organizations, and particularly in high-pressure operational environments such as EKAB, performance appraisal should not be confined to a formal administrative procedure. Instead, it should function as a mechanism for empowerment, skill development, and professional support. International experience demonstrates that appraisal systems linked to training opportunities, guidance, and targeted staff development enhance job satisfaction, organizational commitment, and overall effectiveness [1,3,12]. The findings of the present study support this approach, as the newer appraisal system was perceived as more useful and supportive of employees’ professional development. These results underline the importance of strengthening performance appraisal systems through targeted policy interventions. In particular, the implementation of structured training programs for both evaluators and employees could enhance understanding, consistency, and acceptance of the appraisal process [3,12]. In addition, the standardization of feedback procedures may improve transparency and perceived fairness, ensuring that performance evaluations are aligned with clear criteria and developmental goals [5,9]. Such policy measures could reinforce the role of appraisal systems not only as administrative tools, but also as mechanisms for professional growth, motivation, and quality improvement within emergency healthcare services.

Limitations

The present study has certain limitations that should be considered when interpreting its findings. First, the cross-sectional design does not allow for causal inferences, but only for the identification of associations between the variables examined. Consequently, causal relationships between employee characteristics, knowledge, perceptions, and levels of satisfaction with the performance appraisal process cannot be established.

In addition, data were collected through self-reported questionnaires, which may be subject to social desirability bias or recall bias. Although anonymity and confidentiality were ensured, the possibility that participants’ responses reflected personal attitudes or perceptions rather than their actual experiences cannot be fully excluded.

Another limitation concerns the level of familiarity with the newer performance appraisal system introduced by Law 4940/2022, which was at a relatively early stage of implementation at the time of data collection. Limited exposure to and understanding of the new framework may have influenced participants’ responses, particularly regarding knowledge and perceptions, and these findings may evolve as the system becomes more established.

Finally, although the sample included employees from a large number of EKAB regional branches, the findings cannot be readily generalized to all healthcare organizations or other public sector bodies. Organizational context, institutional culture, and administrative practices may differ substantially across settings, potentially limiting the external validity of the results.

## Conclusions

This study identified clear differences in knowledge, perceptions, and job satisfaction among EKAB employees regarding two consecutive performance appraisal systems. Although the earlier appraisal system was more familiar to employees, the newer system was associated with more positive perceptions and higher levels of satisfaction, indicating that perceived fairness, transparency, and functionality outweigh familiarity with the system itself. The findings underscore that effective performance appraisal systems should be characterized by clear criteria, consistent application, meaningful feedback, and active employee involvement. In high-intensity emergency prehospital care settings such as EKAB, appraisal processes should function as tools for professional development, empowerment, and continuous improvement rather than as mechanisms of formal control. These results provide evidence-based insights that may support the design and implementation of more effective performance appraisal policies within public healthcare organizations.
